# Editorial: Defining a healthy microbiome

**DOI:** 10.3389/frmbi.2025.1652331

**Published:** 2025-07-18

**Authors:** Blaž Stres

**Affiliations:** ^1^ D13 Department of Catalysis and Chemical Reaction Engineering, National Institute of Chemistry, Ljubljana, Slovenia; ^2^ Department of Animal Science, Biotechnical Faculty, University of Ljubljana, Ljubljana, Slovenia; ^3^ Faculty of Civil and Geodetic Engineering, Institute of Sanitary Engineering, Ljubljana, Slovenia

**Keywords:** gut microbiome, microbial diagnostics, microbiome-host interactions, microbial memory, public and one health, dysbiosis, microbiome-based interventions, environmental microbial exposure

This collection of five articles from *Frontiers in Microbiomes* provides a diverse yet interconnected exploration of the gut microbiome, microbial dynamics in animal and human health, and the potential clinical and public health applications of microbiome science.

The first study by Oliver et al. focused on characterizing intestinal dysbiosis through the use of a stool microbial test called TestUrGut^®^. The study aimed to evaluate the clinical utility of this test by analyzing 154 patients with digestive discomfort. The researchers investigated the abundance of key microbial markers using quantitative polymerase chain reaction (qPCR). They found significant correlations between microbial profiles and clinical features such as bowel patterns, BMI, and gender. For example, *Akkermansia muciniphila* was more abundant in women and those with lower BMI, while variations in *Methanobrevibacter smithii* and Firmicutes/Bacteroidetes ratios aligned with symptoms of diarrhea or constipation. Importantly, their dysbiosis index successfully distinguished pathological dysbiosis from transient imbalances. The study concluded that microbial markers offer valuable clinical insights, supporting the use of stool microbiota analysis as a diagnostic aid for gastrointestinal disorders.

Yiu et al. investigated the association between flagellin-producing gut microbiota and high-density lipoprotein cholesterol (HDL-C) levels in humans. The study analyzed two population-based cohorts: the 500 Functional Genomics Project (500FG) and a Chinese cohort. The authors found sex-specific correlations between flagellated bacteria and HDL-C levels. In 500FG women, higher flagellum-producing capacity correlated with increased HDL-C, mainly driven by species such as *Roseburia intestinalis* and *R. inulinivorans*. In the Chinese cohort, this correlation was stronger in males, with key contributors including *Eubacterium rectale*. Proteomic analysis showed that human liver tissue expresses TLR5 and can detect diverse flagellins, suggesting a mechanistic link through which flagellins promote ApoA1 production and thus HDL-C synthesis. However, not all flagellated bacteria conferred the same benefit, highlighting the importance of species-specific and contextual factors such as diet and geography. The authors emphasized that flagellated microbiota partially explain interindividual differences in HDL-C levels and point to possible microbiome-based interventions for cardiovascular health.

Hay et al. explored chicken caecal enterotypes in indigenous Kadaknath and commercial Cobb lines and their associations with *Campylobacter* abundance. By sampling 600 chickens from 60 farms in western India, they identified three enterotypes: PA1 (Firmicutes-dominant with high *Faecalibacterium*), PA2 (low diversity with high *Phascolarctobacterium* and *Campylobacter*), and PA3 (Bacteroidota-dominant). PA2 was most associated with elevated *Campylobacter* levels, posing food safety and public health risks. The study demonstrated that farming practices, including biosecurity measures, flock management, and environmental factors, significantly influence enterotype distribution. Random Forest models identified predictors such as farm type (open vs. enclosed), visitor policies, and the presence of other animals. These findings suggest that modifying farming practices could help reduce pathogen carriage and improve poultry health and food safety.

Zorgani and Das presented an opinion piece in which they discussed the concept of gut microbiome memory. They proposed that the microbiome retains a form of ecological and immunological memory shaped by early exposures, diet, infections, and environmental interactions. This memory then influences future microbial responses and host physiology. The authors cited evidence such as rapid microbial adaptation to dietary changes, sustained shifts in metabolic potential following nutrient exposure, and enhanced resistance to pathogens after prior infections. They also discussed emerging research on bacterial memory, including *E. coli*’s swarming memory linked to iron levels, and noted that antibiotic exposure during early life might disrupt microbial memory, contributing to long-term disease risks. The article advocates for further research into microbiome memory mechanisms to inform personalized interventions and therapies.

Finally, Asumang et al. reviewed the role of microbial dynamics in promoting health and immune function in school settings. The authors emphasized that while hygiene practices in schools aim to prevent infection, excessive cleanliness may reduce exposure to beneficial microbes, potentially contributing to increasing rates of allergies and autoimmune diseases—a concept rooted in the hygiene hypothesis. The authors explored how microbial exposure in school environments could shape immune system development, citing the importance of maternal microbiota, birth mode, and feeding practices. The review highlighted studies showing that diverse environmental microbes support immune training, whereas dysbiosis can increase disease susceptibility. The authors advocated for balanced hygiene policies and microbial interventions (e.g., probiotics, prebiotics, or environmental enrichment with beneficial microbes) to enhance health outcomes in schools.

In summary, these articles collectively illustrate the multifaceted roles of microbiota across species, settings, and stages of life. The contributions underscore the potential of microbiome-based diagnostics (Oliver et al.), highlight mechanistic links between microbial functions and systemic health markers (Yiu et al.), demonstrate the influence of farming practices on animal microbiomes and pathogen risk (Hay et al.), and propose innovative frameworks such as microbial memory (Zorgani and Das) that could reshape our understanding of host-microbiome interactions. Finally, Asumang et al. called attention to the need for reconsidering hygiene and microbial exposure in schools to foster long-term health. These insights point toward a new era in which tailored microbiome management plays a central role in medicine, agriculture, and public health—particularly when approached systemically by integrating microbiome multi-omics data with environmental descriptors, enhancing our awareness of the thermodynamic boundaries defining a healthy microbiome.

